# The Burden of Pediatric Asthma

**DOI:** 10.3389/fped.2018.00186

**Published:** 2018-06-22

**Authors:** Giuliana Ferrante, Stefania La Grutta

**Affiliations:** ^1^Department of Science for Health Promotion and Mother and Child Care, University of Palermo, Palermo, Italy; ^2^National Research Council of Italy, Institute of Biomedicine and Molecular Immunology, Palermo, Italy

**Keywords:** asthma, burden, children, cost, epidemiology, prevalence, morbidity, mortality

## Abstract

Asthma is the most common chronic disease in children, imposing a consistent burden on health system. In recent years, prevalence of asthma symptoms became globally increased in children and adolescents, particularly in Low-Middle Income Countries (LMICs). Host (genetics, atopy) and environmental factors (microbial exposure, exposure to passive smoking and air pollution), seemed to contribute to this trend. The increased prevalence observed in metropolitan areas with respect to rural ones and, overall, in industrialized countries, highlighted the role of air pollution in asthma inception. Asthma accounts for 1.1% of the overall global estimate of “Disability-adjusted life years” (DALYs)/100,000 for all causes. Mortality in children is low and it decreased across Europe over recent years. Children from LMICs particularly suffer a disproportionately higher burden in terms of morbidity and mortality. Global asthma-related costs are high and are usually are classified into direct, indirect and intangible costs. Direct costs account for 50–80% of the total costs. Asthma is one of the main causes of hospitalization which are particularly common in children aged < 5 years with a prevalence that has been increased during the last two decades, mostly in LMICs. Indirect costs are usually higher than in older patients, including both school and work-related losses. Intangible costs are unquantifiable, since they are related to impairment of quality of life, limitation of physical activities and study performance. The implementation of strategies aimed at early detect asthma thus providing access to the proper treatment has been shown to effectively reduce the burden of the disease.

## Introduction

Asthma is the most prevalent chronic respiratory disease worldwide, affecting more than 300 million people of all ethnic groups throughout all ages (1). It is the most common chronic disease in children, imposing an increasingly consistent burden on health system (2). Despite the various asthma phenotypes described in children, this condition is overall recognized as a chronic inflammatory disease of the airways characterized by variable symptoms of wheeze, breathlessness, chest tightness and/or cough associated with expiratory airflow limitation that may resolve spontaneously or in response to medication (3).

The understanding of the global burden of pediatric asthma has increased over the last two decades thanks to national and international studies on general populations. In defining asthma prevalence, epidemiological surveys have focused on self-reported (or parent-reported) symptoms by using standardized questionnaires, rather than on doctor diagnosis.

In particular, wheezing has been considered the most important symptom in the identification of asthma (4). The International Study of Asthma and Allergies in Childhood (ISAAC) consisted of 3 phases. Phase I was conducted during the period 1991–1995 in children and adolescents aged 6–7 and 13–14 years, respectively, in 56 countries throughout the world (5); Phase III, including 237 centers in 98 countries, was performed 5 years later, in order to examine changes in prevalence of symptoms after Phase I (6). Data on respiratory health in the younger age group were obtained from questionnaires fulfilled by parents, whereas the older age group was able to self-complete the questionnaires, which were constructed in order to be accessible to people from different countries and income levels. This accurate methodology explains the high response rate with 721.601 children involved in Phase One and 1.187.496 in Phase Three. The Italian part of ISAAC, the SIDRIA (Italian acronym for Studi Italiani sui Disturbi Respiratori nell'Infanzia e l'Ambiente) Study, was performed in two different phases between 1994 and 2002 and provided information on the epidemiology of childhood asthma in Italy, basing on questionnaires including the ISAAC core module (7, 8). Data collected from these surveys contributed to improve knowledge in the epidemiology of childhood asthma through a standardized approach. A survey to examine variations in prevalence rates of childhood asthma, wheeze and wheeze with asthma in Europe has been recently carried out (9), but further studies are needed for a more comprehensive and ongoing assessment of disease prevalence, morbidity and hospitalization all over the world (10).

## Global and time trends of asthma in children

**Figure 1:**
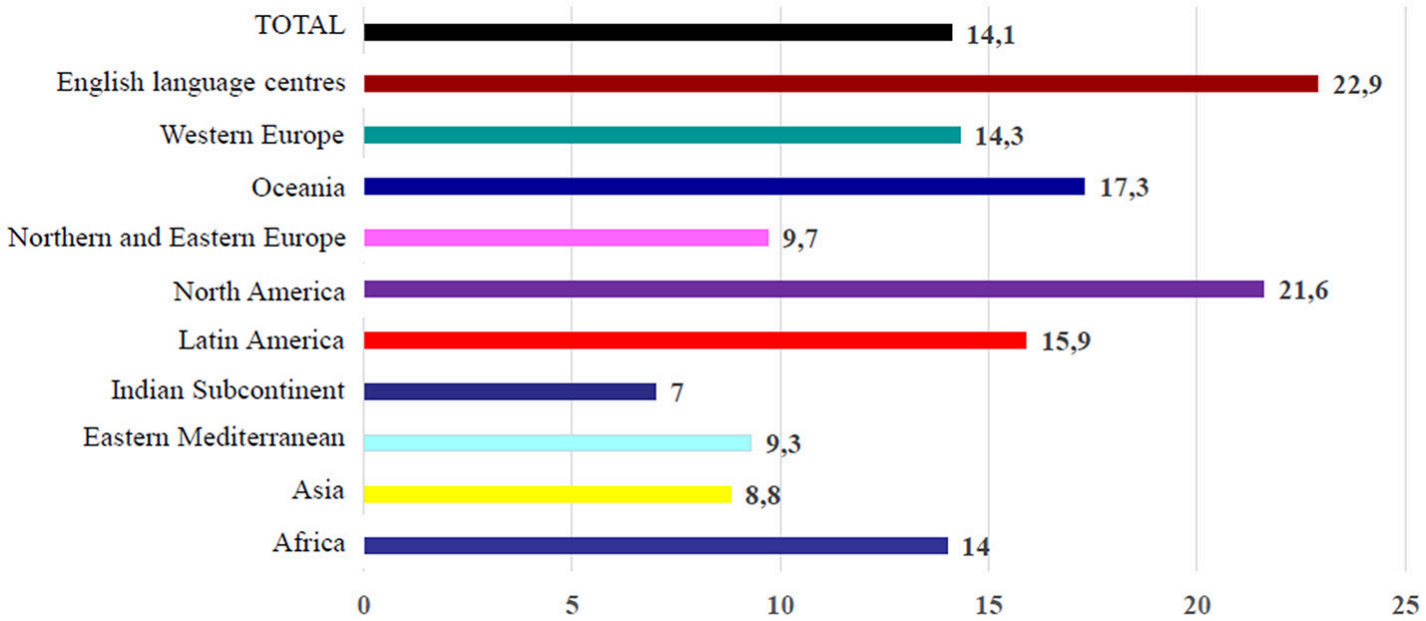
Prevalence of current wheezing in adolescents 13–14 years: data from ISAAC Phase III.

Overall, “asthma ever” was more common in High-Income Countries (HICs), even though the highest prevalence of severe symptoms among children with current (past 12 months) wheeze were found in Low- and Middle-Income Countries (LMICs) (Figure 1) (11). Conspicuous variations in the lifetime prevalence of asthma have been reported between countries all over the world (6). Between ISAAC Phase One and Phase Three prevalence of asthma symptoms became globally increased in children and adolescents ranging from 11.1 to 11.6% and from 13.2 to 13.7%, respectively (12). Generally, prevalence of “asthma ever” significantly increased in children and adolescents, probably due to a greater awareness of this condition and changes in diagnostic practice. However also host (genetics, atopy) and environmental factors (microbial exposure, exposure to passive smoking, and air pollution), seemed to contribute to the observed increasing trends. In particular, lifetime prevalence of asthma remained the same or even decreased in HICs, whereas it increased in many LMICs, especially in Eastern European as well as in Latin American and Northern African countries (6, 13). In Europe, increased prevalence has been reported in children (12).

Between SIDRIA Phase One and Phase Two, prevalence of “asthma ever” tended toward a stabilization in children, ranging from 9.1 to 9.5%, whereas in adolescents it increased from 9.1 to 10.4%. Prevalence of “current wheeze” resulted to be little increased in children, ranging from 7.8 to 8.6%, whereas it slightly decreased in adolescents, ranging from 10.5 to 9.7%. Noteworthy, asthma prevalence in adolescents increased only in those living in large metropolitan areas, with a percent prevalence change of 3.3% (14). Prevalence of asthma symptoms in migrant children was found to increase with the number of years of living in Italy, with risk of lifetime asthma and current wheeze very similar to Italian children (15). This finding adds further evidence to the critical role that environmental exposures may play in the development of asthma in childhood. Lastly, prevalence of asthma was found to be higher in males as in children as in adolescents, whereas asthma was more common in girls after puberty. These gender differences might be attributed to a narrower airways caliber in males than females in early life due to the effects of different hormonal factors (7).

## Risk factors for asthma in childhood

### Genetics and epigenetics

Differences in asthma prevalence observed in vary ethnic groups all over the world may be explained by differences in the genetic susceptibility. Although the specific contribution of genetics to asthma has not thoroughly clarified, a large number of genetic markers reliably associated with asthma and airway inflammation have been identified to date. Particularly, different polymorphisms in the 17q21 locus have been found to be associated with asthma. This is considered one of the strongest loci for asthma, even though its function is still not known. Risk alleles of some single nucleotide polymorphisms (SNPs) in this locus were associated with an increased number of CD4+ cells as well as with the number of eosinophils in the airway wall biopsies of asthmatics, suggesting the involvement of these genes in the Th2 pathway in asthma (16). A new locus associated with time to asthma onset was identified at position 16q12 (17). A recent study found novel genetic factors to potentially explain sex-specific asthma effects during childhood (18). Moreover, a SNP on chromosome 8 was associated with early lung function decline (19). No specific models of genetic transmission have been identified so far.

A multi-factorial model characterized by complex relationships between genes and environment has been instead proposed. In this context, greater and greater interest on epigenetics has been focused in recent years, finding that environmental exposures are able to modulate genes expression in a complex interaction that may be even transferred from mother to child (20). More recently, traffic related air pollution's influence on asthma was showed to be associated with DNA demethylation of a CpG site in the promoter region of the Ten-eleven translocation 1 gene, a possible biomarker for childhood asthma (21).

### Atopy

Data from epidemiological studies showed the strong link between asthma and atopy. Indeed, the family history of atopy is considered one of the most relevant risk factors for developing asthma. The relationship between allergic sensitization and asthma onset has been well-documented (22, 23). However, the causal relationship between individual allergen exposure and the development of symptoms has been not clearly delineated, probably due to the complexity of interactions between timing and dose of exposure and genetic predisposition. Almost 60% of schoolchildren with asthma are allergic, mainly to perennial allergens such as house dust mites, animal dander and molds. These indoor allergens play an important role in eliciting asthma exacerbations, by increasing airway inflammation and bronchial hyper-responsiveness (24). Lastly, it was recently showed that aeroallergen sensitization before the age of 5 years significantly increased the risk of asthma with persistence into adolescence (25).

### Microbial exposure

A reduced microbial exposure since early life through improved sanitation and increased rates of immunization have been linked to the increased prevalence of asthma observed in childhood. Changes in environment and/or lifestyle have been suggested to alter the development of the immune system increasing the risk of asthma in genetically susceptible subjects, basing on the so called “hygiene hypothesis.” Accordingly, children raised in modern environment with a scanty natural microbial burden may be prone to develop allergic diseases in view of an under-stimulation of the immune system. Indeed, recent evidences showed that exposure to some microbes can protect from atopy, whereas others seem to promote allergic diseases. The timing of exposure to as well as the properties of the infectious agent, in addition to the genetic susceptibility of the host, may influence the future development of asthma (26). There is evidence for a critical role of respiratory virus infections in early life for asthma development. In particular, Respiratory Syncytial Virus (RSV) and Human Rhinovirus (HRV) are most frequently associated with wheezing episodes in preschool children and with asthma development in the next years. Approximately 50% of the infants with lower respiratory tract infections caused by RSV during the first 12 months of life developed persistent asthma at school age (27). In children at high risk for development of asthma, HRV-induced wheezing in infancy resulted to be the strongest predictor of doctor diagnosed asthma at age 6 years (28, 29). In addition to viral causes, some studies suggested that infections by atypical bacterial, such as *Mycoplasma pneumoniae* and *Chlamydia pneumonia*, may play a role in inducing and exacerbating asthma (30, 31).

### Environmental exposure

The increased asthma prevalence in metropolitan areas with respect to rural ones and, overall, in industrialized countries, highlighted the role of air pollution in asthma inception (14). Exposure to both outdoor and indoor pollutants has been associated with increased asthma exacerbations, rates of hospitalization and reduced lung function (32–34). Even though a cross-sectional study on 5 European birth cohorts recently showed no associations between air pollutants exposure and asthma prevalence (35), a European population-based birth cohort study on more than 14,000 children found that increasing exposure to nitric dioxide (NO_2_) and particulate matter with a diameter of less than 2.5 μm (PM_2.5_) at the birth address was associated with increased asthma incidence at age 14–16 years (36). Further evidence comes from a meta-analyses of birth cohort studies showing that increased childhood exposure to PM_2.5_ and black carbon was associated with increased risk of asthma at age 12 years (37). The most dangerous environmental exposure in children derives from Environmental Tobacco Smoke (ETS), which is universally recognized as a major risk factor for asthma. Exposure to prenatal and early postnatal passive smoke may have adverse effect on both the immune system and the structural and functional development of the lung; this may explain the subsequent increased risk of incident asthma (38). Exposure to ETS at school age is associated with an increased risk of severity and exacerbations and may be considered a risk factor for asthma persistence in later life (39). In recent years increasing interest raised the novel concept of Third-Hand Smoke (THS), the combination of tobacco smoke pollutants which remain in an indoor environment after tobacco has been smoked. Since infants and children are prone to the risks related to THS exposure, investigations are warranted to study the health effects of THS relevant to different exposure pathways and profiles occurring also in pre-natal life (40). Lastly, a new threat to the respiratory health of children and adolescents has been raised from the spread of electronic cigarettes, the most commonly used tobacco product among adolescents (41). Recent studies have shown an association with asthma symptoms in adolescents (42, 43). Further research on the health effects of electronic cigarettes is advised.

## The global burden of disease

### Lifelong outcomes

Asthma can appear at any stage throughout life, but it generally develops in childhood (44). Data from the Melbourne Asthma Study reported that 47% of individuals with persistent asthma, and even 75% of subjects classified with severe asthma, at age 6 still had asthma symptoms at age 50 years (45). Noteworthy, children with severe asthma were those at increased risk of developing Chronic Obstructive Pulmonary Disease (COPD) (45–47). Therefore, asthma can be considered a lifelong disease with a major burden especially in subjects suffering from severe asthma.

### Morbidity

In 1990, the Global Burden of Disease Study (GBD) proposed the “Disability-adjusted life years” (DALYs) as a measure of disease burden. DALYs quantify how many years of life are lost due to death and/or non-fatal illness or impairment. This health gap measure can be considered as the sum of two components: years of life lost plus years lived with disability (YLDs) (48). The latter measure is calculated as the prevalence of each disease sequela multiplied by the disability weight for that sequela. Asthma was the 14th highest ranked cause of global YLDs at all ages, but specific data for children were not available (49). In the GBD 2015, it accounted for 1.1% of the overall global estimate of DALYs/100,000 for all causes. Overall, asthma represents the second most important respiratory disease after COPD when considering the burden of disease as measured by both YLDs and DALYs.

### Mortality

Mortality for asthma is relatively low at all ages. In Europe, asthma is responsible for 0.4% of all deaths (43,000 persons), with wide differences among countries (50). In the GDB 2015, a decrease of 26.7% was observed in comparison with 1990. The decrease in age-standardized death rates was 58.8% between 1990 and 2015. The greatest decrease was observed in HICs, reflecting a better access to health services as well as better treatment options following international guidance (1). Asthma mortality in children is low and is significantly associated with symptoms prevalence and hospital admissions (51). Hence, when comparing childhood asthma mortality between countries, any reduction in prevalence has to be taken into account. Over recent years, asthma mortality in children decreased across Europe, with little difference between countries. This would be attributable to a better control of symptoms due to improvements in treatment of asthma attacks together with the more widespread use of inhaled corticosteroids which have been shown to reduce mortality at all ages (52). Noteworthy, data from the National Review of Asthma Deaths Confidential Enquiry Report showed that in United Kingdome 80% of asthma deaths occurred in people with poor adherence to treatment and in those who had taken more bronchodilators (53).

## Socio-economic cost of childhood asthma

Asthma is a chronic condition that can assume different severity degrees throughout patient's life, with significant social impact and economic burden. In fact, this disease can be associated with limitations on physical and social aspects of daily life of children and their caregivers, especially when symptoms are not controlled (3). Overall, global asthma-related costs are high and significantly vary across countries, depending on several factors, such as the type of health system, financial resources on Public Health and methods of data collection (54).

**Figure 2:**
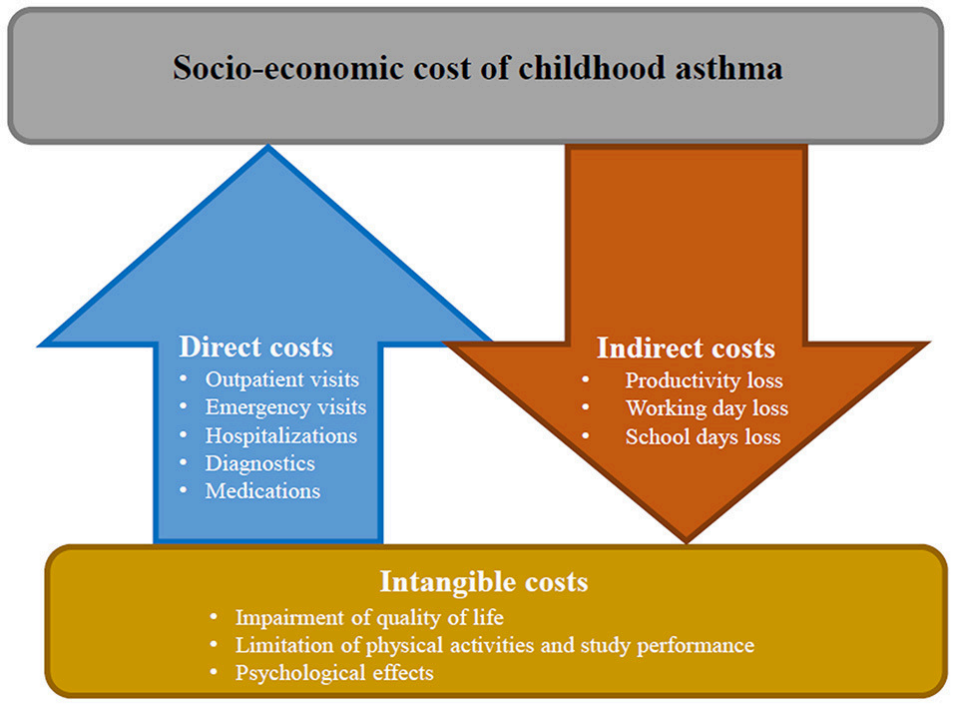
Socio-economic cost of childhood asthma: direct, indirect, and intangible costs.

Usually, asthma-related costs are classified into direct, indirect and intangible costs (Figure 2). Direct costs generally account for 50–80% of the total costs and include: disease management (e.g., outpatient visits, visits to emergency services, hospital admission, medications), complementary investigations or treatment and other costs (e.g., assistance in home care, transportation to medical visits) (54, 55). In children and adolescents with asthma the number of outpatient visits as well as the number of visits to emergency services is higher than in non-asthmatics, increasing according to the disease severity degree (56). Asthma is one of the main causes of hospitalization in children which are usually at least twice than in adults. Hospitalizations are particularly common in children aged <5 years with a prevalence that has been increased during the last two decades, mostly in LMICs (57). Medications account for variable costs, which differ across countries depending on health system and public or private insurance coverage (58). Greater use of asthma drugs, particularly inhaled steroids, occurred in recent years globally increased costs related to asthma medications (54).

Indirect costs include work-related losses and early mortality. Although loss of working days is not directly applicable in children, absenteeism from school is a comparable consequence. In childhood asthma indirect costs are usually higher than in older patients: a child with an exacerbation of asthma loses on average 3–5 days school days and at least one of her/his caregivers loses the same working time (59).

Intangible costs are unquantifiable, since they are related to impairment of quality of life, limitation of physical activities, and study performance, with consequent psychological effects such as depression and anxiety. Nonetheless, the social burden of asthma is considerable, not only for the child but also for parents. Therefore, when assessing quality of life in asthmatic children, it is important also to assess the quality of life of the caregivers (52).

## Conclusions

During last decades, asthma prevalence has been increasing worldwide. As a chronic condition that usually starts in early childhood, it imposes a high lifetime burden on individuals, their caregivers and the community. Despite significant progress in health care reached in last decades, there are still consistent disparities between countries. Children from LMICs particularly suffer a disproportionately higher burden in terms of morbidity and mortality (54). The implementation of strategies aimed at early detect asthma thus providing access to the proper treatment has been shown to effectively reduce the burden of the disease (50). The Global Asthma Network (GAN) was established in 2002 as a collaboration between individuals from ISAAC and the International Union Against Tubercolosis and Lung Disease in order to globally improve asthma care through enhanced surveillance, research collaboration, funding and capacity building, access to medical care and quality-assured essential medicines, and education of health professionals and public (60). A survey undertaken by GAN in 120 countries during 2013–2014 showed that only about one in four countries had a national asthma strategy, with a lower proportion in LCMIs than in HICs. In countries with a high prevalence of current wheeze, adopting an asthma strategy was significantly more common than what occurred in low prevalence countries. This may be due to lack of interest or to difficulties in engaging in world-wide epidemiological studies. However, extension of asthma strategies in all countries is strongly recommended, since such an approach could have a big impact on the burden of the disease, by decreasing severity and improving symptoms control (61). In this perspective, asthma, as other non-communicable chronic respiratory diseases, must be included in the agenda of each national authority. It is therefore important that monitoring of prevalence and severity continue globally. Further studies focusing on estimates of asthma costs are warranted, especially in LMICs. Moreover, standard methods of data collection are desirable in order to obtain comparable information from different countries (2). Finally, future asthma research should integrate both pediatric and adult populations in longitudinal studies, with the aim of better understand the role of risk and protective factors on disease onset and severity throughout life (1, 62, 63).

## Author contributions

GF and SL provided substantial contributions to the conception or design of the work, revised the manuscript for important intellectual content, approved the final version, and agreed to be accountable for all aspects of the work.

### Conflict of interest statement

The authors declare that the research was conducted in the absence of any commercial or financial relationships that could be construed as a potential conflict of interest.
